# Development and validation of the multidimensional impacts of movement scale (MIMS) for yoga, weightlifting, and running

**DOI:** 10.3389/fpsyg.2023.1078996

**Published:** 2023-03-01

**Authors:** Sarah Lynn, Julia C. Basso

**Affiliations:** ^1^Department of Human Nutrition, Foods, and Exercise, Virginia Tech, Blacksburg, VA, United States; ^2^Center for Health Behaviors Research, Fralin Biomedical Research Institute at VTC, Roanoke, VA, United States; ^3^School of Neuroscience, Virginia Tech, Blacksburg, VA, United States

**Keywords:** movement, yoga, exercise, lifting, neuropsychology, mental health, proprioception, interoception

## Abstract

**Background:**

Movement is an essential element in maintaining overall well-being, producing both physical and mental health benefits. Yoga is a mindful movement practice, with traditional yogic texts providing a framework, called the Koshas, that delineates how an intentional movement practice may impact multidimensional aspects of an individual. To date, no self-report measure examines the multifaceted ways that movement affects the individual at a physical and psychological level. Therefore, we developed the Multidimensional Impacts of Movement Scale (MIMS) by aligning ancient yogic traditions with current neuroscientific concepts.

**Methods:**

MIMS was developed based on the five categories of the Koshas; 9 questions per Kosha resulted in 45 total questions. Participants (*n* = 103) self-identified as having yoga, running, or weightlifting as their primary movement practice, engaging in this practice at least 30 min per session, once a week, for the past 3 months. Participants engaged in their usual movement practice and then (within 2 h of their workout session) completed the MIMS along with a series of previously validated questionnaires. After a period of 2 weeks, participants completed their normal movement practice once again and took the MIMS a second time to assess test–retest reliability and Cronbach’s alpha. Validity testing included convergent and divergent validity testing through Pearson’s product-moment correlations and confirmatory factor analysis.

**Results:**

One-hundred and three participants completed all study measures. Test–retest reliability demonstrated stability over time (*r* = 0.737, *p* < 0.001). Cronbach’s alpha was between 0.775 and 0.840 for each of the factors, *p* < 0.001. MIMS was sensitive to confirmatory and discriminatory validity testing. Validity was also demonstrated through confirmatory factor analysis (i.e., Chi Square, Comparative Fit Index, Root Mean Square Error of Approximation).

**Conclusion:**

MIMS is a valid and reliable tool to measure the multidimensional impacts of movement. The tool provides information about the effects of movement on a range of physical and psychological elements including subscales representing the body, energy, mind, intuition, and contentment. Physical activities that include aspects of mindfulness may demonstrate the most robust effects on the MIMS.

## Introduction

Physical activity is defined as “any bodily movement produced by skeletal muscle contraction that increases energy expenditure above a basal level”(Piercy et al., 2018). Physical activity is beneficial for a range of physical and mental health issues, including obesity, type II diabetes (Kumar et al., 2019), cancer (McTiernan et al., 2019), and mood and anxiety disorders (Chan et al., 2019), and has been shown to increase the human life span (Anderson and Durstine, 2019). Importantly, exercise produces a range of positive effects at the psychological level including decreased stress, anxiety, and fatigue, and improved energy, mood, self-esteem, and social satisfaction (Sharma et al., 2006; Basso and Suzuki, 2017; Mikkelsen et al., 2017). To date, the majority of research in this realm has focused on either aerobic (e.g., running) or anaerobic (e.g., weight lifting) exercise. Yoga, however, is a mindful physical activity practice that incorporates movement, breathwork, concentration, and meditation (Rakel, 2012; Govindaraj et al., 2016). A 2012 National Health Interview Survey (NHIS) found that approximately 31 million (13.2%) US adults have tried yoga in their lifetime and about 21 million (8.9%) US adults practice yoga regularly (Cramer et al., 2016) The NHIS found that yoga practitioners were motivated to practice yoga due to wellness and disease prevention, increased energy, enhanced immune function, and reduced stress, and in a comparison review of the health benefits of yoga vs. traditional aerobic exercise (Ross and Thomas, 2010), yoga was found to be as effective as exercise at reducing stress and enhancing mood, motor, and cognitive performance (Taheri et al., 2018).

Considering the range of exercise-induced psychological effects, measuring such outcomes is challenging, and to assess outcomes, researchers often utilize a battery of self-reported measures and neuropsychological tasks, which take time and expertise to administer. Importantly, no self-report scales exist to measure the multidimensional outcomes of movement, and no known scales address the complex system of outcomes of mindful movement practices such as yoga. Currently, two validated tools exist to assess yoga. First, the Beliefs about Yoga Scale (Sohl et al., 2011) was developed to aid researchers in finding participants likely to complete longitudinal yoga studies. The information gathered from the Beliefs about Yoga Scale illuminates positive and negative beliefs about yoga’s potential outcomes and its connection to spiritual traditions. Second, the Yoga Self Efficacy Scale (Sohl et al., 2011) was developed to determine how people feel during the practice of yoga. Questions on this scale target body, breath, mind, and confidence in knowing how and what to do during a yoga class. Based on the lack of scales to assess yoga and exercise more generally, we sought to develop and validate a scale to assess the multidimensional outcomes of movement. We intentionally chose to develop this scale using both a yogic and neuroscientific framework as the developers of this scale were an experienced meditation teacher (with >10,000 h of teaching experience) and a PhD neuroscientist who specializes in how mind–body-movement practices affect neuropsychological functioning.

**Figure 1:**
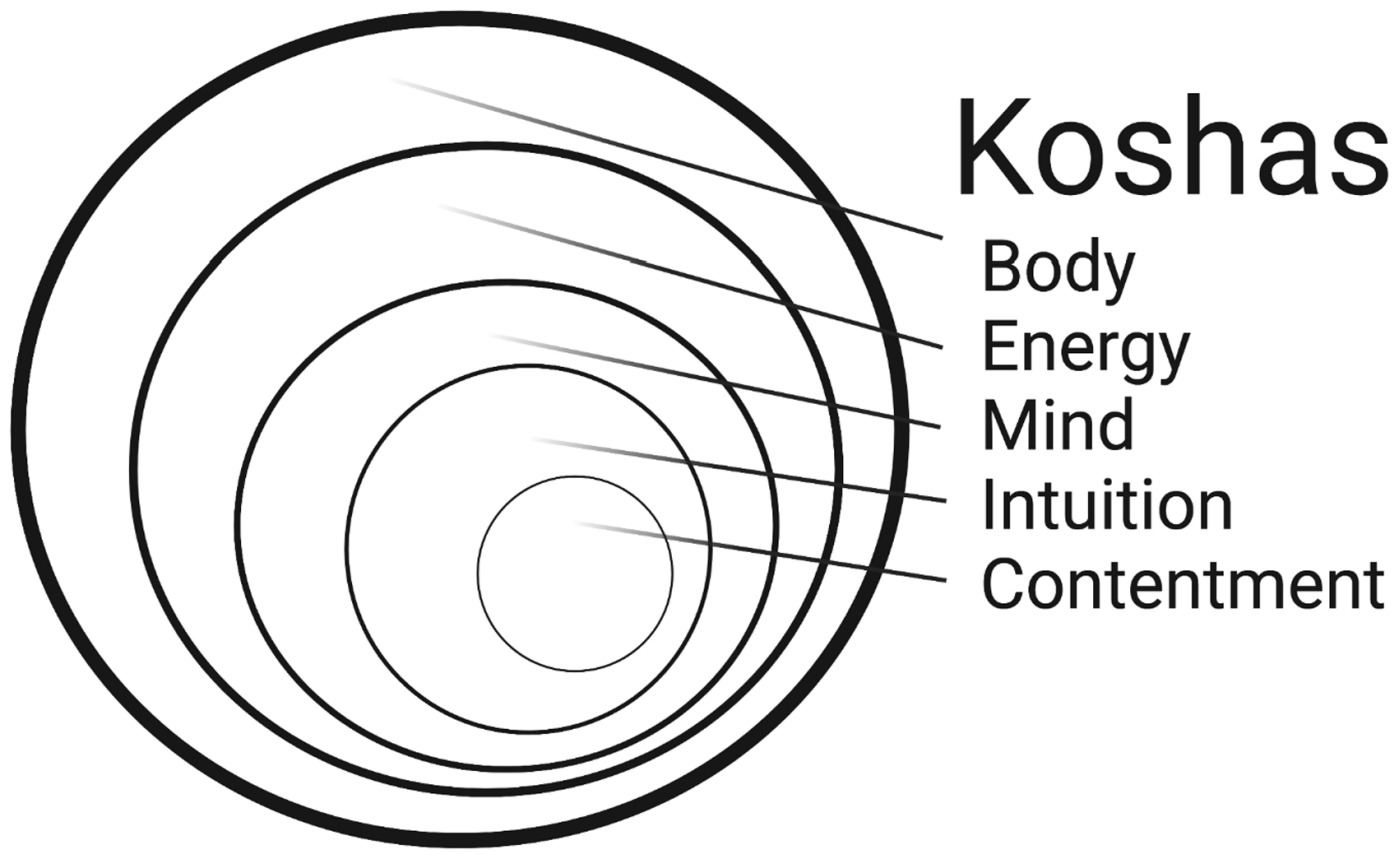
Diagram demonstrating the concentric nature of the koshas.

In regard to the yogic framework, the Yoga Sutras of Patanjali (Patañjali and Zambito, 1992), a primary yogic text, explains a process of achieving freedom through yoga, including ethical considerations (*yamas* and *niyamas*), movement (*asana*), breathwork (*pranayama*), sensory control (*pratyahara*), concentration (*dharana*), meditation (*dhyana*), and the resulting freedom (*samadhi*). In addition, the Taittiriya Upanishads (200 CE), a book on the nature of life, death, love, and divine presence, explains a system of multidimensionality among layers of every individual called the Koshas (Easwaran, 2007). The five layers of the Koshas are the body, energy, mental function, wisdom, and contentment. The Koshas are often spoken of as containers such that the physical body contains the energetic body, which further contains thoughts, emotions, and sense perceptions (i.e., the mind), which contains wisdom (i.e., intuition), which holds contentment at the core (Figure 1). In regard to the neuroscientific framework, we examined the Koshas in the context of neuroscience and found excellent alignment between each Kosha and a particular psychological/neuroscientific construct (Table 1). As an example, the Anamaya Kosha (which represents the physical body) aligned with the concepts of proprioception, balance, and embodiment, whereas the Pranayama Kosha (which represents the breath and energetic life force) aligned with the concepts of vitality and fatigue. The Koshas representing mind, intuition, and contentment were each paired with respective psychological constructs (Table 1).

**Table 1:** Overlapping yogic and neuroscientific concepts regarding the multidimensional aspects of being.

Overlapping yogic and neuroscience concepts		
8 Fold Path of Patanjali – The steps to doing yoga	Koshas – Multidimensional Aspects of being	Neuroscience/Psychology
1. *Yamas* 2. *Niyamas* Ethical restraint and development	No Kosha equivalent	Not assessed in present study
3. *Asana* Physical poses	*Anamaya Kosha* Physical body [BODY]	Proprioception, balance, embodiment
4. *Pranayama* Breathing practices	*Pranayama Kosha* Breath, force, energy [ENERGY]	Vitality, fatigue
5. *Pratyahara* Control of the senses	*Manomaya Kosha* Thoughts, emotions and sense perception [MIND]	Aspects of mental health (anxiety, depression, and affect)
6. *Dharana* Concentration 7. Dhyana Meditation	*Vjnanamaya Kosha* Wisdom [INTUITION]	Confidence, trust, compassion, interoception
8. *Samadhi* Freedom from constraint	*Anandamaya Kosha* Bliss and contentment [CONTENTMENT]	Satisfaction, awe, gratitude

From this theoretical framework, we developed and subsequently validated a tool based on aligned yogic and neuroscientific concepts that assessed the multifaceted impacts of movement (i.e., body, energy, mind, intuition, contentment; Table 1). We utilized three different movement practices [i.e., yoga (balance/flexibility/mindfulness); running (aerobic); and weightlifting (anaerobic)] to test the hypothesis that the Multidimensional Impacts of Movement Scale (MIMS) is valid and reliable using rigorous statistical analytic techniques.

## Methods

### Procedure

The Virginia Tech Institutional Review Board (IRB) approved this study (IRB-21-074). MIMS was created in four phases: (1) item generation; (2) review by a panel of experts; (3) focus groups, and (4) testing. The study authors are experts in behavioral neuroscience and yoga and completed the initial item generation through conversations that surrounded mapping the Koshas onto modern neuroscientific concepts. We utilized a panel of experts to review the first iteration of MIMS, including a neuroscientist, a yoga instructor, and two experts in tool validation. Several iterative revisions were made considering changes from this panel. Feedback from two focus groups, including both undergraduate and graduate students at Virginia Tech, helped to further refine the individual items and study format.

**Figure 2:**
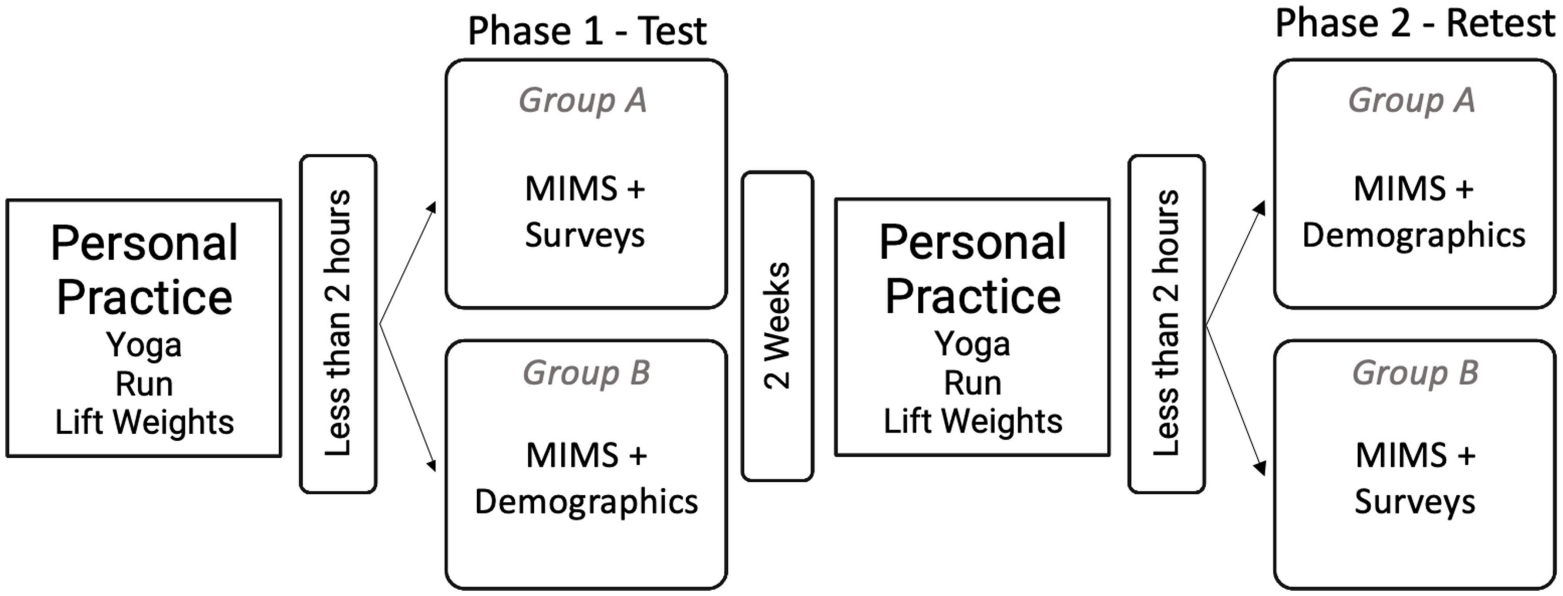
Study design, including study elements and timeline.

Recruitment occurred through social media posts, online posts hosted through the university, and flyers hung around campus. Direct emails were also sent to related places of business (e.g., gyms and yoga studios). After passing a screening questionnaire, participants were randomized into Group A (received MIMS + surveys for validation at test, and MIMS + demographics at retest) or Group B (received MIMS + demographics at test, and MIMS + surveys for validation at retest; Figure 2). The random division into two groups served to minimize any effect the surveys had on responses to MIMS. Participants completed their usual movement practice, and within 2 h completed their initial test/survey. We chose this 2-h period as the acute effects of exercise are most potent up to 2 h after exercise cessation (Basso et al., 2015). After a 2-week wash-out period, participants were instructed to complete their typical workout again and then complete their retest within 2 h. Participants were instructed that this 2nd workout should be as close to the initial as possible in terms of type of activity, length and intensity of workout, and time of day completed. Participants were compensated $20 for completing the entire study with no partial payments.

### Participants

A total of *n* = 146 participants volunteered and completed screening. Participants were included if they were 18 years or older, had English as their primary language, and self-identified as having yoga, running, or weightlifting as their primary form of movement practice for greater than three months. Participants were excluded if they did not pass the Physical Activity Readiness Questionnaire for Everyone (PAR-Q+; Warburton et al., 2011) or reported that their regular movement sessions lasted less than 30 min. Of the 146 participants who took the screening tool, 24 did not meet the eligibility criteria. Of the 122 participants who started the study, 19 did not complete all necessary components of the research and were removed, leaving *n* = 103 participants for analysis.

### Study measures

#### Assessing physical activity readiness

The Physical Activity Readiness Questionnaire (PAR-Q+; Warburton et al., 2011) is a self-report tool created to help individuals make connections between their health and physical activity. The PAR-Q+ was used as a screening tool to assure participants were safe to engage in their regular movement practice. The PAR-Q+ underwent a revision in which some of the questions were revised for clarity. In this study, we use the short form of the PAR-Q+, which has seven questions that can be answered with a “yes” or “no” response. Three questions have space to add greater detail with free writing. From the old to the new version of PAR-Q, there was a strong correlation (*r* = 0.80); the test–retest reliability of the PAR-Q+ is (*r* = 0.99), and it shows a much greater specificity over the PAR-Q.

#### Confirming validity for the *a priori* factor, body

The Activities Specific Balance Confidence Scale (ABCS; Peretz et al., 2006) is a self-report scale providing information about an individual’s fear of falling and confidence in moving through the world. It is a single factor scale with 16 questions, with the overall score reported as an average. Answers to the questions are reported in increments of 10 as confidence percentages that one will not fall given a specific activity. The ABCS validation testing reports Cronbach’s alpha = 0.96 and a test–retest correlation (*r* = 0.92, *p* < 0.001). Convergent Validity was tested against the Physical Self-Efficacy Scale (PSES; Ryckman et al., 1982) while divergent validity was tested with the Positive and Negative Affect Scale (Crawford and Henry, 2004).

The Scale of Body Connection (SBC; Price et al., 2017) is a self-report measure of bodily awareness and dissociation. There are 20 questions divided into 2 subscales: body awareness and body dissociation. The SBC is measured on a 5-Point Likert Scale, with 0 representing “Not at all” and 4 representing “All of the time.” The SBC is best scored using two subscales, with higher scores corresponding to higher levels of body awareness and body dissociation, respectively. An overall score is calculated by reversing the body dissociation score and taking an average of the two subscale scores. The SBC proves reliability with Cronbach’s alpha = 0.83. Construct validity using Structural Equation Modeling found a goodness-of-fit model that demonstrated two independent factors.

#### Confirming validity for the *a priori* factor, energy

The Brief Resilience Scale (BRS; Smith et al., 2008) is a self-report measure of an individual’s perception of resilience. There are six questions and no subscales, with overall score reported as a mean. Questions are answered on a 5-point Likert scale, with 1 representing “Strongly Disagree” and 5 representing “Strongly Agree.” The BRS displays strong internal consistency with a Cronbach’s alpha ranging from 0.80 to 0.91 for each of the four groups used for testing. Principal Component Analysis from all four samples shows only one factor, accounting for 55–67% of the variance. Factor loadings ranged from 0.68 to 0.91.

The Fatigue Severity Scale (FSS; Learmonth et al., 2013) is a self-report measure bringing together emotional and physical symptoms of fatigue on one scale. The FSS has nine questions and no subscales, with the overall score reported as an average. It is measured on a 7-point Likert scale with 1 representing “Strongly Disagree” and 7 representing “Strongly Agree.” The FSS was validated using the Intraclass Correlation Coefficient (ICC) with 95% confidence intervals and a test–retest score of 0.751. Convergent validity of the FSS and the Modified Fatigue Impact Scale (MFIS) (Larson, 2013) show *r* > 0.5 Spearman Correlation.

The Subjective Vitality Measure (SVM; Ryan and Frederick, 1997) is a self-report measure of an individual’s perception of their vitality or sense of energy and livelihood. Seven questions on this measure are scored on a 7-point Likert Scale with 1 representing “Not at all” and 7 representing “Very True.” Certain items are reverse scored, and the overall score is reported as an average. The SVM earned a Cronbach’s alpha ranging from 0.84 to 0.86 in three samples. The test–retest in both clinical and non-clinical samples was >0.70. Factor analysis revealed eigenvalues = 6.77.

#### Confirming validity for the *a priori* factor, mind

The Beck Anxiety Inventory (BAI; Borden et al., 1991) is a self-report measure of anxiety symptoms, including questions about somatic and psychological experiences related to anxiety. It has 21 questions, with various factor-analytic studies reporting between two to six factors. Questions are asked on a 4-point Likert scale, with 0 representing “Not at all” and 3 representing “Severely – it bothered me a lot.” The total score is calculated by summing responses for each question. Results can be described as 0–21 = low anxiety; 22–35 = moderate anxiety; and 36 and above = potentially concerning anxiety levels. BAI demonstrates high internal consistency with Cronbach’s alpha = 0.91 with median item correlations at *r* = 0.56. Principal Components Analysis (PCA) with eigenvalues greater than 1.0 with a varimax rotation converged in 19 iterations, resulting in five factors, which accounted for 60% of the variance.

The Beck Depression Inventory (BDI; Beck et al., 1988) is a self-report measure of depression symptoms. BDI has 21 questions, and factor analysis over 25 years of re-testing shows between three and seven factors. BDI includes multiple-choice questions, instructing the participant to select the phrase that best describes them (e.g., “I do not feel sad,” “I feel sad,” “I am sad all the time and I cannot snap out of it,” or “I am so sad and unhappy that I cannot stand it”). The responses are rated from 0 to 3, and it is scored as a sum of all responses. These sums are then rated as: 1–10 = these ups and downs are considered normal; 11–16 = mild mood disturbance; 17–20 = borderline clinical depression; 21–30 = moderate depression; 31–40 = severe depression; and over 40 = extreme depression. BDI’s reliability shows a Cronbach’s alpha = 0.86 in the clinical population and 0.81 in non-clinical populations. The test–retest reliability showed *r* > 0.60. Concurrent Validity with Hamilton Psychiatric Rating Scale for Depression (HRSD; Miller et al., 1985) showed *r* = 0.72–0.73 for clinical populations and *r* = 0.60–0.74 in nonclinical populations.

The Positive and Negative Affect Scale (PANAS; Crawford and Henry, 2004) is a self-report measure of positive and negative affect. PANAS has 20 questions with two subscales: positive affect and negative affect. It is scored on a 5-Point Likert Scale, with 1 representing “Very slightly or not at all” and 5 representing “Extremely.” Both positive and negative affect scores range from 10 to 50, with higher scores representing higher levels of that particular affective state. PANAS has a Cronbach’s alpha = 0.89 for Positive Affect and 0.85 for Negative Affect. Confirmatory factor analysis showed both models of good and poor fit.

#### Confirming validity for the *a priori* factor, intuition

The Compassion Scale (CS; Pommier et al., 2020) is a self-report measure of one’s kindness and desire to lessen the suffering of others. CS includes 16 items divided among four subscales: kindness, common humanity, mindfulness, and indifference (reverse scored), with the overall score and subscales reported as averages. A variety of studies show CS to be reliable, with Cronbach’s alpha ranging from 0.77 to 0.90. Test–retest reliability demonstrated *r* = 0.81. Known group validity showed marked differences, as expected in meditators vs. non-meditators, and Structural Equation Modeling found a good fit with three positive subscales and one negative subscale.

The Metacognition Questionnaire-30 (MCQ-30; Wells and Cartwright-Hatton, 2004) is a 30 question self-report measure of cognitive confidence. The MCQ-30 has five subscales: confidence, positive beliefs about worry, cognitive self-consciousness, negative beliefs about uncontrollability and danger, and need to control thoughts. A 4-Point Likert scale is used in the MCQ-30, with 1 representing “Do not agree” and 4 representing “Agree very much.” Summation scores range from 30 to 120, with higher scores representing higher levels of unhelpful metacognitions. Cronbach’s alpha for MCQ-30 ranges from 0.70 to 0.93 for each of the five subscales.

The Multidimensional Assessment of Interoceptive Awareness (MAIA; Mehling et al., 2012) is a self-report measure of an individual’s awareness of their internal sensations. It has 32 questions with 8 subscales: noticing, not-distracting, not-worrying, attention regulation, emotional awareness, self-regulation, body listening, and trusting. MAIA uses a 6-Point Likert Scale with 0 = Never to 5 = Always. Scores are calculated as the average of each domain with selected items reversed. Internal Consistency ranged from 0.66 to 0.82 for individual subscales of MAIA. Correlations among subscales ranged from 0.09 to 0.60. The validity of MAIA was tested with convergent and divergent scales.

#### Assessing validity for the *a priori* factor, contentment

The Dispositional Positive Emotions Scale (DPES; Shiota et al., 2006) contains a subscale measuring Awe. This subscale has been validated individually to measure an individual’s curiosity and wonder about the world (Gottlieb et al., 2018). The Awe Subscale is made up of six questions on a 7-point Likert scale, with 1 representing “Strongly Disagree” and 7 representing “Strongly Agree,” and the overall score reported as an average. The validation study utilized Amazon Mechanical Turk, with participants having >95% approval ratings. Cronbach’s alpha = 0.82 among all six items of the Awe Subscale. The Awe Subscale was validated against other scales considering spirituality and science and was found to have significant and measurable scientific quality.

The Satisfaction with Life Scale (SLS; Pavot et al., 1991) is a self-report measure of subjective well-being. It has five questions and no subscales, scored on a 7-Point Likert Scale with 1 representing “Strongly disagree” and 7 representing “Strongly Agree.” Scores are reported as one total sum, divided into designations of extremely satisfied (31–35), satisfied (26–30), slightly satisfied (21–25), neutral (20), slightly dissatisfied (15–19), dissatisfied (10–14), and extremely dissatisfied (5–9). The SLS proves reliable with a Cronbach’s alpha = 0.85 and test–retest reliability of 0.84. Factor analysis and factor loading were stronger for individual questions than composite scores, ranging from 0.55 to 0.93.

### Power and statistical analysis

An *a priori* power analysis was run using G*Power 3.1 to determine the appropriate number of participants to sufficiently power this study (Faul et al., 2009). We utilized an F test, ANOVA: Repeated measures, within-between interaction using an effect size of 0.25, an alpha error probability of 0.0005 to correct for multiple testing, power level of 0.8, three groups (yoga, running, and weightlifting), two measurements (test vs. retest), correlation among representative measures of 0.5, and nonsphericity correction of 1 to determine a sample size of *n* = 96.

Statistical analysis was completed for validation and reliability of the Multidimensional Impacts of Movement Scale. Cronbach’s alpha and correlations were conducted using SPSS, Version 27.0.1.0, 64-bit edition (IBM SPSS Statistics for Macintosh, 2020). Internal consistency was calculated as Cronbach’s alpha. Pearson’s product–moment correlations were calculated to determine test–retest reliability demonstrating the tool’s stability over time. Convergent and divergent validity were determined with Pearson’s product–moment correlations using previously validated tools alongside the initial test of MIMS. Confirmatory factor analysis (CFA) was completed using RStudio 2022.02.0 Build 443 (R Studio, 2022). CFA was performed to determine if the *a priori* five-factor structure could be confirmed with a good fit model. Scale purification improved model fit through statistical judgment, factor loading, and parsimony to remove any redundant questions based on correlations. Specifically, we utilized an acceptable cutoff for reliability of Cronbach’s alpha >0.700 (Tavakol and Dennick, 2011). As measures of validity are more subjective, we examined the interplay between sample size, factor loading, root mean square error of approximation (RMSEA), and the goodness of fit (Bolarinwa, 2015; Singh, 2017). We then utilized parsimony to simplify and balance the scales. One-Way Analysis of Variance (ANOVAs) was performed to determine statistically significant differences in MIMS outcomes between yogis, runners, and weightlifters; Tukey–Kramer post-hoc analyses were conducted as appropriate. Data are presented as mean (standard error of the mean), and statistical significance was determined using *p* < 0.05 (Table 2).

**Table 2:** Demographic data for all 103 participants in the study.

Basic characteristic	*n*	%	Basic characteristic	*n*	%
*N* = 103					
**Sex**			**Ethnicity**		
Female	82	79.6	Hispanic	7	6.8
Male	21	20.4	Non-Hispanic	95	92.2
**Race**			Prefers not to answer	1	1
Asian	7	6.8	**Education**		
Black	4	3.9	High School	7	6.8
Indigenous	0	0	Some college or vocational training	28	27.2
White	90	87.4	Associates Degree	7	6.8
Prefers not to answer	1	1	Bachelor’s Degree	29	28.2
**Income**			Graduate Degree	32	31.1
Low <$40,000	19	18.4			
Middle $40,000–$120,000	36	35		**Mean**	**±SD**
High >$120,000	35	34	**Age**	30.39	12.63
Prefers not to answer	13	12.6			

## Results

### Participants

The initial scale that was developed included 50 total questions, with 10 questions in each of the five *a priori* factors. Through confirmatory factor analysis, we eliminated 1 question from each of the *a priori* factors, leaving a total of 45 total questions (9 per factor). Therefore, the following results are based on these 45 final questions. Final scale and scoring details are available (see Supplementary File 1).

### Reliability

MIMS demonstrated test–retest reliability of *r* = 0.737 with significance of *p* < 0.001. All subscales showed significant stability over time, with *r* > 0.670, *p* < 0.001 or higher for each subscale. Internal consistency was confirmed with Cronbach’s alpha for each factor and individual question. There were nine questions in each of the five *a priori* factors, which were all examined individually. All questions remained, showing that a removal of any question would not result in a change in Cronbach’s alpha below 0.700. Cronbach’s alpha is between 0.775 and 0.840 for each of the factors (body *α* = 0.781, energy *α* = 0.840, mind *α* = 0.815, intuition *α* = 0.775, and contentment *α* = 0.830).

### Validity

The body factor was positively associated with the SBC awareness (*r* = 0.509, *p* < 0.001) and negatively associated with dissociation (*r* = −0.296, *p* = 0.002) subscales. No significant association was found with the ABCS.

The energy factor was negatively associated with the FSS (*r* = −0.226, *p* = 0.022) and SVM (*r* = 0.602, *p* < 0.001). No significant association was found with the BRS.

The mind factor was negatively associated with the BAI (*r* = −0.218, *p* < 0.027) and BDI (*r* = −0.392, *p* < 0.001), positively associated with PANAS positive affect (*r* = 0.428, *p* < 0.001), and negatively associated with PANAS negative affect (*r* = −0.339, *p* < 0.001).

The intuition factor was positively associated with the CS (*r* = 0.377, *p* < 0.001) and MAIA (*r* = 0.580, *p* < 0.001). No significant association was found with the MCQ-30.

The contentment factor was positively associated with the DPES awe subscale (*r* = 0.515, *p* < 0.001) and the SLS (*r* = 0.461, *p* < 0.001).

### Confirmatory factor analysis

**Figure 3:**
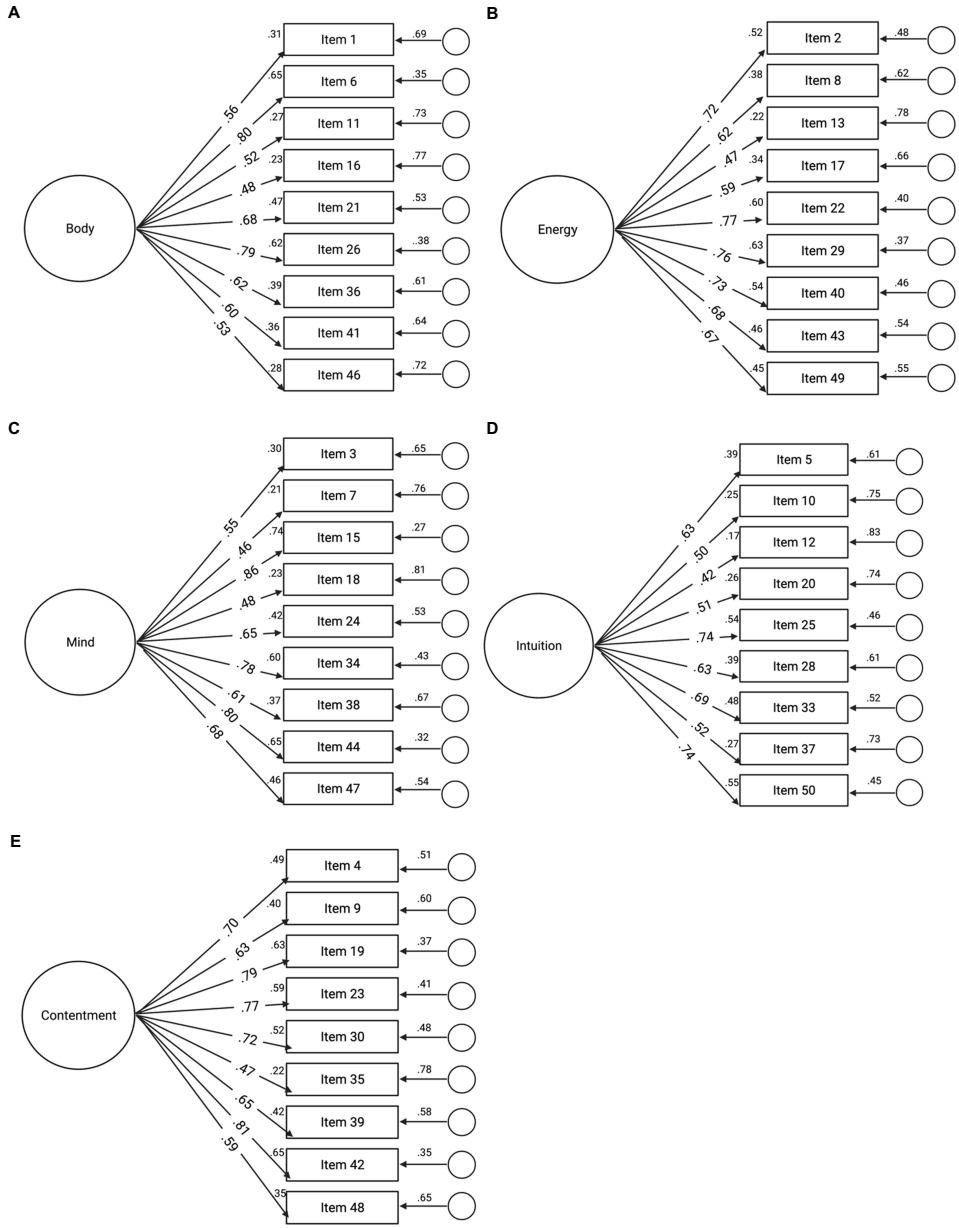
**(A-E)** Confirmatory factor analysis (CFA) model for five factors of MIMS. Note that items in each of the five factors are from the original 50 items. Based on confirmatory factor analysis, one item was removed from each factor. Final items can be found in Supplementary File 1.

Confirmatory factor analysis supported five distinct factors. Scale purification was completed based on initial data. After reviewing correlations, factor loading, and to improve parsimony, items 14 (contentment), 31 (body), 32 (energy), 27 (mind), and 45 (intuition) were removed from MIMS. Specifically, item 14 on the contentment scale had the highest factor load. When analysis was run without item 14, Cronbach’s alpha improved for that subscale, and the other factor loadings adjusted to create an overall better model fit. In order to balance the scales, individual items with the highest factor loading were removed, which improved both the overall model fit and Cronbach’s alpha for each subscale. The revised scale has 45 items, 9 in each factor. The data are represented in Table 3 and Figures 3A–E.

**Table 3:** Confirmatory factor analysis data.

Models	Chi Square	df	CFI	RMSEA
(*N* = 103)				
Factor 1 Body	40.835	27	0.971	0.071
Factor 2 Energy	30.352	27	0.995	0.035
Factor 3 Mind	73.096	27	0.934	0.129
Factor 4 Intuition	41.539	27	0.962	0.073
Factor 5 Contentment	98.015	27	0.917	0.161

### Differences among movement groups

**Figure 4:**
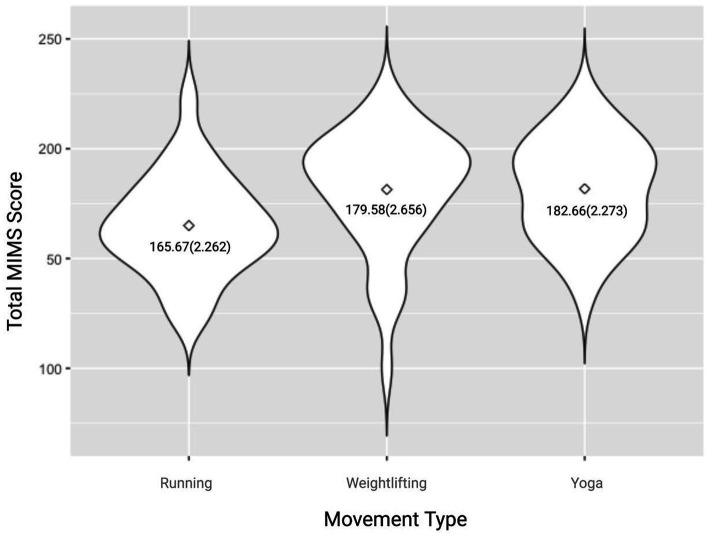
Violin plot of Total MIMS scores by movement group. Data reported as mean (SEM).

Regarding the overall MIMS score, statistically significant differences were found between the three movement groups [*F*(2, 100) = 4.095, *p* = 0.020], with this effect being driven by body [*F*(2, 100) = 5.618, *p* = 0.005] and intuition [*F*(2, 100) = 4.083, *p* = 0.020]. Regarding the MIMS total score, the yoga group reported the highest score while the running group reporting the lowest score (Table 4; Figure 4). Post-hoc analyses revealed that the yoga group scored significantly higher than the running group on the total MIMS score [16.991, 95% CI (2.29 to 31.69), *p* = 0.19], as well as the body [3.791, 95% CI (0.92 to 6.67), *p* = 0.006] and intuition [3.546, 95% CI (0.38 to 6.71), *p* = 0.024] subscales. Additionally, the weightlifting group scored significantly higher on the body [3.370, 95% CI (0.50 to 6.24), *p* = 0.017] and intuition [3.177, 95% CI (0.02 to 6.34), *p* = 0.049] subscales than the running group.

**Table 4:** Between group differences for yoga, running and weightlifting.

	Yoga Mean (SEM)	Running Mean (SEM)	Weightlift Mean (SEM)	*F*	*p*-Value
*N* = 103					
Body	38.42 (0.437)	34.63 (0.475)	38.00 (0.505)	5.618	0.005
Energy	36.24 (0.514)	33.37 (0.514)	35.50 (0.629)	2.149	0.122
Mind	36.32 (0.520)	33.07 (0.493)	35.13 (0.612)	2.678	0.075
Intuition	35.84 (0.510)	32.30 (0.479)	35.47 (0.557)	4.083	0.020
Contentment	36.32 (0.507)	33.81 (0.590)	35.29 (0.632)	1.438	0.242
Total	182.66 (2.273)	165.67 (2.262)	179.58 (2.656)	4.095	0.020

## Discussion

In this study, we delineated the process for validating the Multidimensional Impacts of Movement Scale (MIMS), which included item generation, examination of the items/scale through a panel of experts and focus groups, data testing, and validity and reliability analyses. MIMS was built by aligning modern neuroscientific concepts with the traditional yogic framework of the Koshas, which supports the idea that humans are complex beings, with intricate, simultaneous aspects of the self (Easwaran, 2007). Our results demonstrate that the MIMS is valid and reliable with five distinct subscales: body, energy, mind, intuition, and contentment. MIMS is stable over time as represented by strong test–retest scores and demonstrates strong internal consistency with a high Cronbach’s alpha for each of the five distinct subscales, ranging from *α* = 0.775 to 0.840. The tool is valid, showing convergent validity with strong significant correlations between known, previously validated tools, clearly defining the psychological constructs that MIMS measures.

The overall MIMS score indicates the general impact of movement on an individual, while the subscales themselves provide a more nuanced examination of the multidimensional outcomes of movement. The body subscale measures an individual’s awareness and control over their body. A high score on the body subscale indicates high levels of physical awareness and low levels of bodily dissociation. The energy subscale measures vitality and an individual’s ability to turn energy into action. A high score on the energy subscale indicates increased levels of vitality and decreased levels of fatigue. The mind subscale measures the integration of thoughts, emotions, and senses. A high score on the mind subscale indicates high levels of positive affect and low levels of negative affect (e.g., depression, anxiety). The intuition subscale measures how much an individual trusts their thoughts and emotions to guide decision-making. A high score on the intuition subscale indicates high levels of interoceptive awareness and compassion. Finally, contentment measures the ease and satisfaction an individual feels within oneself and the world around them. A high score on the contentment subscale indicates high levels of awe and satisfaction with life.

We recommend that MIMS can be used in movement research, both for scientific and clinical purposes. Importantly, the tool will reduce participant burden by having one scale with various outcomes. The self-report element of this tool makes it easy to implement, taking only a few minutes to complete. This tool will allow consistency of measurement across different movement modalities and may even be implemented in other mind–body-movement techniques such as meditation. MIMS can also be applied within the movement industry as a tool to assess outcomes of group and individual exercise, helping individuals or businesses to visualize the results of their movement practice/offerings.

### The effects of yoga, weightlifting, and running on MIMS

As the Physical Activity Guidelines for Americans (Piercy et al., 2018) encourage participation in cardiorespiratory, strength training, and flexibility/balance activities weekly, the tool was intentionally validated across these three movement categories (i.e., running, weightlifting, and yoga). Our data indicate that different forms of movement may produce different outcomes at the physical and psychological levels. Therefore, encouraging multiple movement forms across the week may create the most balanced results across the full range of MIMS outcomes. Specifically, yoga practitioners scored highest on the MIMS indicating that yoga may impact more elements measured by this scale than weightlifting or running. Post-hoc analyses revealed that yoga practitioners scored higher than runners on total MIMS as well as body and intuition subscales, and weightlifters scored higher than runners on body and intuition subscales. We hypothesize that these findings may be because yoga is a mindfulness-based technique that incorporates aspects of the physical body (*asana*) as well as breathwork (*pranayama*) and meditation (*dhyana*). Additionally, weightlifting has been considered as a contemplative practice and may incorporate mindful aspects as intense focus and concentration are needed to safely lift heavy weights (Vernon, 2018). These types of physical activities that incorporate multiple aspects of physical and mental wellbeing may be optimal to enhance overall wellness.

Similar to our results, others have demonstrated that yoga may provide additional benefits beyond aerobic exercise. Specifically, a systematic review and meta-analysis of 22 randomized controlled trials found that compared to active controls, yoga improved lower limb strength, lower body flexibility, and depression levels (Sivaramakrishnan et al., 2019). Another integrative review found that yoga is more beneficial than aerobic exercise for reduction of anxiety symptoms (Cole et al., 2022). Others have shown that yoga may be more helpful than aerobic exercise in terms of executive functioning (e.g., attention, working memory; Moore et al., 2019), though some studies have demonstrated equivalent results (Telles et al., 2013; Vhavle et al., 2019). Conversely, other work revealed that physical exercise is more beneficial at improving social self-esteem compared to yoga (Telles et al., 2013). In regard to comparisons between aerobic and anaerobic training, a recent study during the COVID-19 pandemic found that individuals practicing aerobic exercise had lower levels of depression and anxiety than those practicing anaerobic exercise (i.e., strength training), but individuals who practiced both had better levels of health perception than either group (da Costa et al., 2022). Such discrepancies in the literature may be due to the fact that yoga and other physical activities are not standardized, with the protocols significantly varying between studies (e.g., acute vs. long-term; different lengths of the intervention; different assessment tools). MIMS will allow future studies to have a standardized assessment tool to determine the multidimensional outcomes of movement including aspects of body, energy, mind, intuition, and contentment.

### Limitations and future directions

While the study shows strong reliability and validity, there are some limitations to this research. First, we utilized a convenience sample with the population being mostly white (87.4%), female (79.6%), and young (mean age 30.4 years). Therefore, outcomes would benefit from sampling a more diverse population. Second, participants engaged in diverse workout experiences. Controlling for the same time of day, duration, and intensity of workouts may further refine outcomes. Third, this research was conducted during the COVID-19 pandemic. We did not control for pandemic-based variables such as wearing a mask during workouts, previous or current COVID-19 status, or other aspects of the pandemic. Closer consideration to pandemic variables may be warranted in future studies.

Future research with the MIMS is needed to investigate the influence of a range of movement practices including dance, tai chi, qi gong, swimming, or cross-training. Additionally, researchers may be interested in utilizing MIMS for team sports such as soccer, football, basketball, baseball, lacrosse, or rugby. Future research may also seek to investigate the relationship between the MIMS outcomes and brain-based effects using tools such as electroencephalography or magnetic resonance imaging. Researchers may also consider investigating the influences of exercise duration, exercise habits, age, and COVID-19 considerations on MIMS outcomes. Finally, cultural considerations should be made through culturally sensitive translations into other major languages, allowing the tool to be used more broadly.

MIMS should be used as a standard tool when investigating the outcomes of movement practices, particularly when investigating mind–body impacts. As the original framework of this scale is rooted in yogic texts designed to explore and explain the multidimensional aspects of any individual, MIMS may help explain varied outcomes of movement among individuals. MIMS can also help individuals find their desired results and motivations for movement as the scale may help identify unexpected positive effects of movement. Professionals may use MIMS to help guide individuals to their most needed movement practice.

## Conclusion

MIMS is a valid and reliable tool that measures the multidimensional impacts of movement. Test–retest reliability confirms stability over time (*r* = 0.737). Cronbach’s alpha is between 0.775 and 0.840 for all five factors. Confirmatory Factor Analysis demonstrates a good model fit for each factor along with convergent and divergent validity creating specificity in what the tool measures. MIMS can be used in research, the fitness industry, or by individuals.

## Data availability statement

The raw data supporting the conclusions of this article will be made available by the authors, without undue reservation.

## Ethics statement

The studies involving human participants were reviewed and approved by Virginia Tech Institutional Review Board. The patients/participants provided their written informed consent to participate in this study.

## Author contributions

JB and SL conceptualized the study. SL ran all study procedures and analyzed all data and wrote the first version of the manuscript and created all figures and tables. JB edited the manuscript to produce the final version. All authors contributed to the article and approved the submitted version.

## Funding

JB is an Integrated Translational Health Research Institute at Virginia (iTHRIV) Scholar, supported by the National Center for Advancing Translational Science of the National Institutes of Health Award UL1TR003015/KL2TR003016.

## Conflict of interest

The authors declare that the research was conducted in the absence of any commercial or financial relationships that could be construed as a potential conflict of interest.

## Publisher’s note

All claims expressed in this article are solely those of the authors and do not necessarily represent those of their affiliated organizations, or those of the publisher, the editors and the reviewers. Any product that may be evaluated in this article, or claim that may be made by its manufacturer, is not guaranteed or endorsed by the publisher.
