# Supplementary material for: Development and validation of the multidimensional impacts of movement scale (MIMS) for yoga, weightlifting, and running
Source: Front Psychol. 2023 Mar 1;14:1078996. doi: 10.3389/fpsyg.2023.1078996 (PMC10014715; doi:10.3389/fpsyg.2023.1078996)
Supplement: Supplementary file 1 [file Table_1.pdf]

Multidimensional Impacts of Movement Scale (MIMS)

Respond to these statements with one mark per row.

Consider how your movement practice influences how you feel now, in this moment. While you may cross-train some, think about the movement practice that you most identify with as your primary movement practice.

	No Answer 0	Disagree 1	2	3	4	Agree 5
1. I am in control of my physical balance.						
2. I am thriving.						
3. I am able to learn new things.						
4. I am at ease.						
5. I am able to pay attention to the way my mind works.						
6. I am coordinated.						
7. I have an easy time remembering things.						
8. I am able to focus on the task at hand.						
9. I am comfortable with the unexpected.						
10. I make choices based on increasing the greater good.						
11. I am physically strong.						
12. I am not obligated to act as others expect.						
13. I am open.						
14. I am able to integrate my thoughts, emotions and senses.						
15. I am flexible.						
16. I am quick to recover.						
17. I can feel emotions in my body.						
18. I am whole.						
19. I am confident of my memories.						
20. I am able to release tension in my body.						
21. I am alert.						
22. I see beauty all around me.						
23. I am able to let go of fears.						
24. I fully understand who I am.						

Multidimensional Impacts of Movement Scale (MIMS)

Respond to these statements with one mark per row.

Consider how your movement practice influences how you feel now, in this moment. While you may cross train some, think about the movement practice that you most identify with as your primary movement practice.

	No Answer 0	Disagree 1	2	3	4	Agree 5
25. I am able to control my breath.						
26. I am able to observe my life without judgement.						
27. I am breathing easily.						
28. I am healthy in my body.						
29. I notice what I notice.						
30. I am comfortable expressing my feelings.						
31. I want to help others in need.						
32. I am physically able to do the things I want to do.						
33. I am open to the wisdom of the Universe.						
34. I have a full range of emotions.						
35. I am connected to the energy of the Universe.						
36. I am able to replenish my energy.						
37. I am at home in my body.						
38. I am filled with joy.						
39. I feel free of fatigue.						
40. I am aware of my bodily sensations.						
41. I am able to experience the physical sensations of emotions.						
42. I am comfortable with my own mistakes.						
43. My conscience is clear.						
44. I naturally know what to do next.						
45. I trust my intuitions.						

Scoring MIMS

- To determine ***subscale scores***, add the values for each question indicated below.
- For a ***total MIMS score***, add together the subscale scores.

Higher scores indicate a greater influence of movement.

	Score
**Body**<br>1, 6, 11, 15, 20, 25, 32, 37, 41	
**Energy**<br>2, 8, 13, 16, 21, 27, 36, 39, 44	
**Mind**<br>3, 7, 14, 17, 23, 30, 34, 40, 42	
**Intuition**<br>5, 10, 12, 19, 24, 26, 29, 33, 45	
**Contentment**<br>4, 9, 18, 22, 28, 31, 35, 38, 43	
**Total**<br>Maximum 225	

Body
Awareness of and control over one's body
1. I am in control of my physical balance.
6. I am coordinated.
11. I am physically strong.
15. I am flexible.
20. I am able to release tension in my body.
25. I am able to control my breath.
32. I am physically able to do the things I want to do.
37. I am at home in my body.

Energy
Vitality and ability to turn energy into action
2. I am thriving.
8. I am able to focus on the task at hand.
13. I am open.
16. I am quick to recover.
21. I am alert.
27. I am breathing easily.
36. I am able to replenish my energy.
39. I feel free of fatigue.
44. I naturally know what to do next.

Mind
Integration of thoughts, emotions, and senses
3. I am able to learn new things.
7. I have an easy time remembering things.
14. I am able to integrate my thoughts, emotions, and senses.
17. I can feel emotions in my body.
23. I am able to let go of fears.
30. I am comfortable expressing my feelings.
34. I have a full range of emotions.
40. I am aware of my bodily sensations.
42. I am comfortable with my own mistakes.

Intuition
Trust in how thoughts and emotions guide decision-making.
5. I am able to pay attention to the way my mind works.
10. I make choices based on increasing the greater good.
12. I am not obligated to act as others expect.
19. I am confident of my memories.
24. I fully understand who I am.
26. I am able to observe my life without judgement.
29. I notice what I notice.
33. I am open to the wisdom of the Universe.
45. I trust my intuitions.

Contentment
Ease and contentment with oneself and the world around them
4. I am at ease.
9. I am comfortable with the unexpected.
18. I am whole.
22. I see beauty all around me.
28. I am healthy in my body.
31. I want to help others in need.
35. I am connected to the energy of the Universe.
38. I am filled with joy.
43. My conscience is clear.
